# Successful regression of newly formed corneal neovascularization by subconjunctival injection of bevacizumab in patients with chemical burns

**DOI:** 10.3389/fmed.2023.1210765

**Published:** 2023-06-22

**Authors:** Wen-yan Peng, Li-wen He, Xiao-fang Yin, Bin-Bing Zhou, Tao Zhou, Shi-you Zhou

**Affiliations:** ^1^State Key Laboratory of Ophthalmology, Zhongshan Ophthalmic Center, Sun Yat-sen University, Guangdong Provincial Key Laboratory of Ophthalmology and Visual Science, Guangdong Provincial Clinical Research Center for Ocular Diseases, Guangzhou, China; ^2^Department of Ophthalmology, The Second People's Hospital of Foshan, Foshan, China; ^3^Department of Ophthalmology, The Affiliated First Hospital of Guangdong Pharmaceutical University, Guangzhou, China; ^4^Department of Ophthalmology, First People’s Hospital of Guiyang, Guiyang, China

**Keywords:** chemical burns, corneal neovascularization, bevacizumab, ocular surface reconstructions, wound healing

## Abstract

**Purpose:**

To investigate the effect and timing of subconjunctival bevacizumab injection on inhibiting corneal neovascularization (CorNV) in patients after chemical burns.

**Methods:**

Patients with CorNV secondary to chemical burns were involved. Two subconjunctival injections of bevacizumab (2.5 mg/0.1 mL per involved quadrant) with an interval of 4 weeks were administered, and followed up a year. The area occupied by neovascular vessels (NA), accumulative neovascular length (NL), mean neovascular diameter (ND), best-corrected visual acuity (BCVA) and intraocular pressure (IOP) were evaluated. Complication was also recorded.

**Results:**

Eleven patients with CorNV were involved. Eight patients had a history of surgery (four had amniotic grafts, one had keratoplasty, and three had amniotic grafts and keratoplasty). Decreasing in NA, NL, and ND were statistically significant at each time point compared to the baseline (*p <* 0.01). CorNV that developed within 1 month was considerably regressed, and vessels with fibrovascular membranes were found to be narrower and shorter than pretreatment. BCVA improved in five patients (from one to five lines), remained unchanged in five patients, and decreased in one patient compared to pretreatment.

**Conclusion:**

Subconjunctival bevacizumab injection has a particular potential for the regression of CorNV, especially newly formed within 1 month in patients after chemical burns.

## Introduction

Corneal neovascularization (CorNV) can lead to limbal stem cell deficiency, significant visual impairment, and subsequent high-risk keratoplasty with poor prognosis (1). The current methods for treating CorNV include the use of steroids (2), laser photocoagulation (3), fine needle diathermy (4), and photodynamic therapy (5). The effects of these treatments are variable in humans.

Recently, neutralization of vascular endothelial growth factor (VEGF) has been demonstrated to effectively inhibit angiogenesis and promote corneal graft survival in animal models (6). In addition, topical and subconjunctival administration of the anti-VEGF agent bevacizumab (Avastin), as an off-label treatment, has been used to inhibit CorNV in patients with herpes simplex keratitis (7), lipid keratopathy (8), corneal transplantation (9), bullous keratopathy (9), Stevens–Johnson syndrome (10), and pterygium (11). In another study, subconjunctival administration of bevacizumab was found to effectively inhibit CorNV in the rabbit corneal alkali burn model (12). It was also shown that aflibercept was superior to bevacizumab in a rat chemical burn-induced neovascularization model (13); however, there was no CorNV regression after treatment with aflibercept, betamethasone, or their combination (14) in the rabbit corneal alkali burn model. Results generated in animal models have been inconsistent, and clinical observations have revealed that neovascular vessels do not disappear in most patients receiving bevacizumab treatment. A previous study has suggested that topical bevacizumab treatment can improve the overall graft success rate by preventing neovascularization (15).

Some authors have suggested that early application of anti-VEGF agents may be a better approach for preventing CorNV. However, no study has revealed a definitive intervention time after chemical burns. Therefore, this study aimed to investigate the effect and timing of subconjunctival bevacizumab injection on inhibiting CorNV in patients with chemical burn, and to determine the appropriate timing for its administration.

## Patients and treatments

### Patients

This uncontrolled interventional study was conducted in the Corneal Division of the Zhongshan Ophthalmic Center at Sun Yat-sen University between March 2011 and July 2018. The study protocol conformed to the tenets of the Declaration of Helsinki and was approved by the medical ethics committee of the Affiliated First Hospital of Guangdong Pharmaceutical University (202263). Informed consent was obtained from all patients before the study began.

The inclusion criteria for enrollment were as follows: (1) a history of ocular surface burns, (2) superficial or stromal CorNV extending >2 mm from the limbus, (3) surgical or no surgical intervention, and (4) with a minimal interval of 2 weeks from local dexamethasone inefficacious. The exclusion criteria were as follows: (1) current or recent (≤3 months) corneal and/or ocular surface infection (with bacteria, viruses, fungi, or Acanthamoeba), (2) a history of any other treatment with anti-VEGF agents (topical or systemic), and (3) pregnancy, uncontrolled hypertension, and a history of thromboembolic events.

Eleven consecutive patients (nine men and two women) who met the above criteria were enrolled in this study. The age range of the patients was 18–48 years (mean age = 32.64 ± 10.30 years). Among these patients, three had not undergone any surgery before treatment, one had a history of corneal transplantation, four had a history of amniotic membrane transplantation (AMT), and three had a history of both corneal transplantation and AMT. The interval between the occurrence of the patients’ chemical burns and enrollment in this study varied from 1 to 180 months (mean = 33.27 ± 51.79 months). According to the medical histories and physical examinations, five patients had developed corneal neovascular vessels within a month after the chemical burn (Table 1. Situation, new), and the other six patients had CorNV more than 1 month after the chemical burn (Table 1. Situation, old). The detailed patient characteristics are listed in Table 1.

**Table 1:** Patient characteristics before and after administration of subconjunctival bevacizumab.

Case	Gender /Age (yr)	Eye	Cause of CNV	Intervals	Pre-treatment	Corneal neovascularization (pre-Inj)						Site of Injection		Effect on CNV (Month 12)			BCVA (logMAR)	
						Range	Location	Situation	NA (mm^2^)	NL (mm)	ND (mm)	First Second		NA (mm^2^)	NL (mm)	ND (mm)	Pre-Inj	Post-Inj
1	F/37	OS	acid burn (hydrochloric)	6 mo	AMT (4 mo)	7–12	deep stromal	old	0.5211	25.1200	0.0336	8, 11	9, 12	0.6827	15.2565	0.0368	1.80	1.00
2	M/48	OD	alkali burn (ammonia)	22 mo	AMT (20 mo); LKP (10 mo); symblepharon separation (6 and 1 mo)	3–9	between graft and recipient bed (deep stromal)	old	0.2543	19.7190	0.0277	4, 8	3, 9	0.1827	17.4523	0.0224	0.70	0. 70
3	M/19	OD	alkali burn (lime)	23 mo	AMT (22 and 21 mo); DALK (20 days)	3–9	beyond graft-host junction (superficial and stromal)	new	0.1290	7.0153	0.0645	4, 8	7	0.0132	0.8327	0.0118	1.00	1.00
4	M/24	OS	acid burn (sulfuric acid)	5 mo	upper eyelid granuloma resection (4 mo)	12–9	superficial and stromal	old	0.5326	33.0391	0.0364	3, 7, 12	3, 6, 12	0.2429	25.5347	0.0262	1.70	1.00
5	M/35	OS	acid burn (sulfuric acid)	5 y	AMT (7 times); symblepharon separation + SK (2 y); DALK (21 days)	1–7	between graft and recipient bed	new	0.2874	9.6677	0.0875	2, 7	3, 9	0.0127	2.8268	0.0135	1.80	1.80
6	M/34	OD	alkali burn (cement)	1 mo	none	1–9	superficial	new	0.9166	59.6203	0.0676	1, 7, 9	1, 7	0.3247	27.7327	0.0298	0.70	0.22
7	F/18	OS	alkali burn (ammonia)	15 y	corneal neoplasm resection + AMT (1 mo)	7–12	superficial and stromal	old	0.2308	17.3568	0.0406	7, 10	6, 11	0.1065	13.5363	0.0254	0.40	0.52
8	M/24	OS	acid burn (hydrochloric)	2 y	none	3–7	deep stromal	old	0.1223	15.8044	0.0296	3, 6	3, 6	0.1326	14.5626	0.0265	0.52	0.52
9	M/34	OS	alkali burn (cement)	3 y	LKP(2 y); PKP(1 y)	7–2	between the graft and the recipient bed	old	0.3433	11.6434	0.0655	2, 7	2, 7	0.2919	12.6768	0.0485	1.30	1.30
10	M/47	OD	alkali burn (lime)	2 mo	pseudopterygium resection + AMT + symblepharon separation (11 days)	5–12	superficial	new	2.1471	45.1907	0.0900	7, 9, 12	6, 9	0.1737	15.2546	0.0213	2.00	0.70
11	M/39	OD	alkali burn (cement)	7 mo	AMT (2 mo)	4–1	superficial	new	1.8343	57.8532	0.0845	4, 7, 12	6, 12	0.2245	15.6277	0.0335	1.22	0.52

F, female; M, male. Intervals: between burn and the first Anti-VEGF treatment. Pretreatment: AMT, amniotic membrane transplantation; LKP, lamellar keratoplasty; DALK, deep anterior lamellar keratoplasty; SK, skin transplantation; PKP, penetrating keratoplasty; Situation: new, developed within one month; old, formed more than one month. Range: Area of CorNV involving clock hours. Effect: CorNV, corneal neovascularization; NA, neovascular area; NL, accumulative neovascular length; ND, mean diameter of neovascular vessels. BCVA, best-corrected visual acuity; logMAR, logarithm of the minimal angle of resolution; Pre-inj., pre-injection; Post-inj., post-injection (Month 12); HM, hand movement; FC, finger count; BE, before eye movement.

### Treatment with Subconjunctival injections of bevacizumab and follow-up

All eyes were administered a subconjunctival injection of bevacizumab after enrollment. Before injection, the ocular surface was anesthetized using proparacaine hydrochloride (Alcon Inc., Fort Worth, TX, United States). Subsequently, bevacizumab was injected subconjunctivally at a site adjacent to the entrance of the CorNV. A dose of 2.5 mg/0.1 mL per involved corneal quadrant was injected to form a conjunctival bulla. After the injection, each patient was treated with 0.3% tobramycin/0.1% dexamethasone eye drops (TobraDex, Alcon Inc.) four times a day for 4 weeks. All eyes received a second subconjunctival injection of bevacizumab in the fourth week after the first injection. After the second injection, 0.3% tobramycin/0.1% dexamethasone eye drops were administered four times a day for the next 4 weeks.

Best-corrected visual acuity (BCVA) and intraocular pressure (IOP) were measured regularly, and slit-lamp biomicroscopy and digital photography were performed before and after treatment at different follow-up visits. IOP was measured using Goldman applanation tonometry. Patients visited at weeks 2, 4, 6, 24, and 48 after the first injection of bevacizumab.

### Evaluation of corneal neovascularization

Corneal photographs were used to evaluate morphological changes in the corneal neovascular vessels. At each visit, at least three front-view corneal photographs (with the light source at an angle of 15° on the left side of the patient) were obtained at magnifications of 10× and 16× using a digital camera (Canon DS126291, Canon Inc., Tokyo, Japan) attached to a slit-lamp microscope (Topcon SL-7E, Topcon Corporation, Tokyo, Japan).

**Figure 1:**
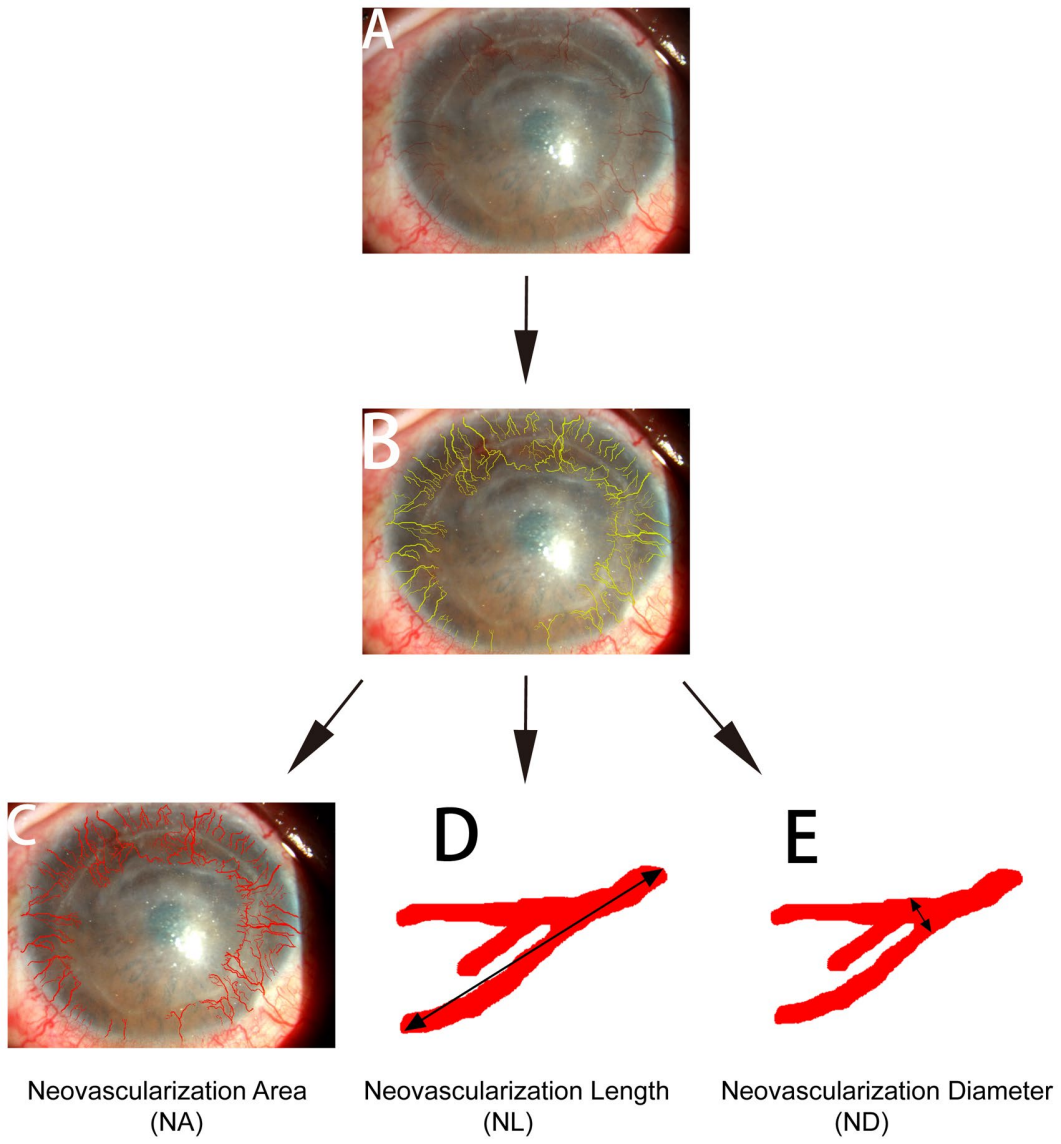
Quantification of corneal neovascular vessels. Front-view corneal photographs **(A)** were analyzed using Adobe Photoshop and Image-Pro Plus 4.0. The total corneal area was identified and measured using Adobe Photoshop, the corneal vessels were manually delineated using the computer’s cursor **(B)**, and the length and diameter of the vessels were determined using Image-Pro. NA **(C)**: neovascular area, the area occupied by the neovascular vessels. NL **(D)**: neovascular length, the approximate cumulative length of all vessels. ND **(E)**: neovascular diameter, the mean diameter of all vessels. When determining NL and ND, only the longest branch and the widest branch were measured for one vessel tree.

The digital slit-lamp corneal photographs were analyzed by a single masked observer using two graphic software applications; Photoshop^®^ CS2 (Adobe Systems Inc., San Jose, CA, United States) and Image-Pro Plus 4.0 (Media Cybernetics, Silver Spring, MD, United States) for Microsoft Windows XP. As described in a previous report (9), the total corneal area was first delineated using Photoshop, and then the corneal vasculature was manually delineated using the computer’s cursor. The area of CorNV and the length and diameter of the neovascular vessels were measured using Image-Pro Plus (Figure 1). The neovascular area (NA) was defined as the area occupied by neovascular vessels and not the involved corneal area. Neovascular length (NL; the approximate cumulative length of all vessels) and neovascular diameter (ND, the mean diameter of all vessels) were obtained by measuring the approximate cumulative length and the mean diameter of all the corneal vessels, respectively (9). When determining the NL and ND, only the longest branch and the widest branch were measured for one vessel tree.

## Statistical analysis

Statistical analysis was performed by Professor Fu-tian Luo from the Department of Biomedical Statistics of the School of Public Health at Sun Yat-sen University. Because of the unknown distribution of the observed data and the small sample size in this study, statistical analysis was performed using the R package nparLD for Microsoft Windows XP, which implements a broad range of rank-based nonparametric methods for analyzing longitudinal data in factorial experiments. The LD-F1 model was applied to compare the whole dataset at different checkpoints. The F1-LD-F1 model was used to compare the changes in new (developed within the first month) and old (noted more than one month ago, and vessels with fibrovascular membranes) vessels (16). A *p* value of less than 0.05 was considered statistically significant. A modified *p* value (modified ANOVA-type statistic) was used for comparisons between two groups, defined as 0.05/the comparison times.

## Results

The patients’ information before and after treatment, is summarized in Table 1. All patients were followed for 1 year after bevacizumab injection. At the last visit, the BCVA was found to have improved in five patients (from 1 to 5 lines), remained unchanged in five patients, and decreased in one patient compared to pretreatment.

### Changes in corneal neovascular vessels

**Figure 2:**
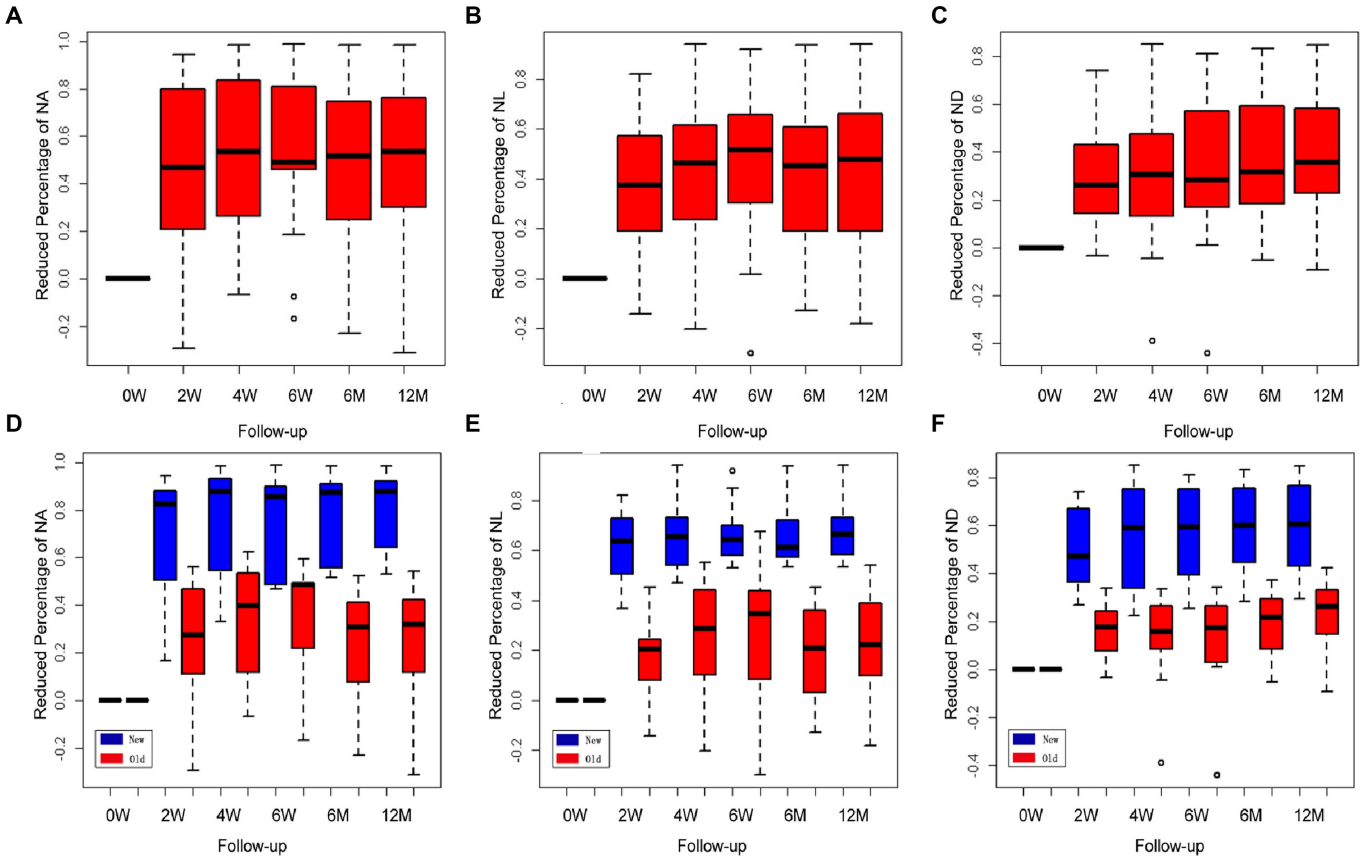
Changes in corneal neovascularization (CorNV) of 11 patients during follow-up. The percent decrease in NA **(A)**, NL **(B)**, and ND **(C)** were described by three box plots. It could reflect the changes relative to the treatment effects at each time point along with the pointwise 95% confidence intervals. Changes in newly developed and more than one-month-old CorNV during follow-up **(D–F)**. Box plots show the percent decreases and relative effects of the three parameters, respectively, at each follow-up time.

The values of the CorNV parameters after treatment were converted into reduced percentages of the baseline (before treatment) values. All data in this study were described using three box plots (Figure 2), and these indicated that the measured distances had skewed distributions. An increase in the median gave rise to a time effect. Compared with the baseline values, the three parameters of area (NA, a and d), length (NL, b and e), and diameter (ND, c and f) decreased significantly (*p* < 0.001) at all checkpoints. By observing the plot along with the pointwise 95% confidence intervals, it was found that the percentages of CorNV improvement increased over time in both groups (Figures 2A–C). Multiple comparisons were performed to investigate the effect between the two contiguous groups. The reduced percentages of NA increased until 6 months after the first injection (24–48 weeks, *p =* 0.27). However, NL and ND reductions peaked at 4 weeks after the first injection (4–6 weeks, for NL *p* = 0.14, for ND *p* = 0.13).

A similar statistical method was used to analyze the different effects between the new and old neovascular vessel groups; the F1-LD-F1 model in the R package was used in this case. Box plots indicated that the measured distances of the three parameters in both groups had somewhat skewed distributions, and the relative effects increased gradually over time. The regression of the new neovascular vessels in five patients was significantly greater than that of the older neovascular vessels in the other six patients either after the first or second injection, and both groups exhibited a time effect in Figures 2D–F (NA, NL, ND, group and time *p* < 0.05). When NA and NL were compared across two contiguous time groups, it was found that the effect of the two groups increased over time until 6 months (24–48 weeks, NA *p* = 0.225; NL *p* = 0.056). The ND decreased until 6 weeks after treatment (6–24 weeks, *p* = 0.134). The regression NA of the new and old neovascular vessels showed a statistically significant difference only after the first injection (0–2 weeks, group *p <* 0.001, other *p* > 0.05). When the NL was examined, it was found that the effects of the two groups were similar at all time points (*p* > 0.05). The regression ND of the two groups was statistically different until 4 weeks (0–2, 2–4 weeks, group *p <* 0.001, other *p* > 0.05).

Among the five patients who had newly formed CorNVs, two patients developed neovascular vessels at the interface between the lamellar corneal graft and native corneal stroma within 1 month after anterior deep lamellar keratoplasty. Four weeks after treatment with subconjunctival bevacizumab, the corneal vessels regressed considerably (Figures 2A–C). Three patients with a history of chemical burns in the previous month exhibited abundant superficial vessels in the peripheral cornea. After treatment, the superficial corneal vessels regressed significantly, and the vessels in the fibrovascular membrane narrowed (Figure 2F). Meanwhile, the corneal edema improved. All patients showed improved BCVA.

**Figure 3:**
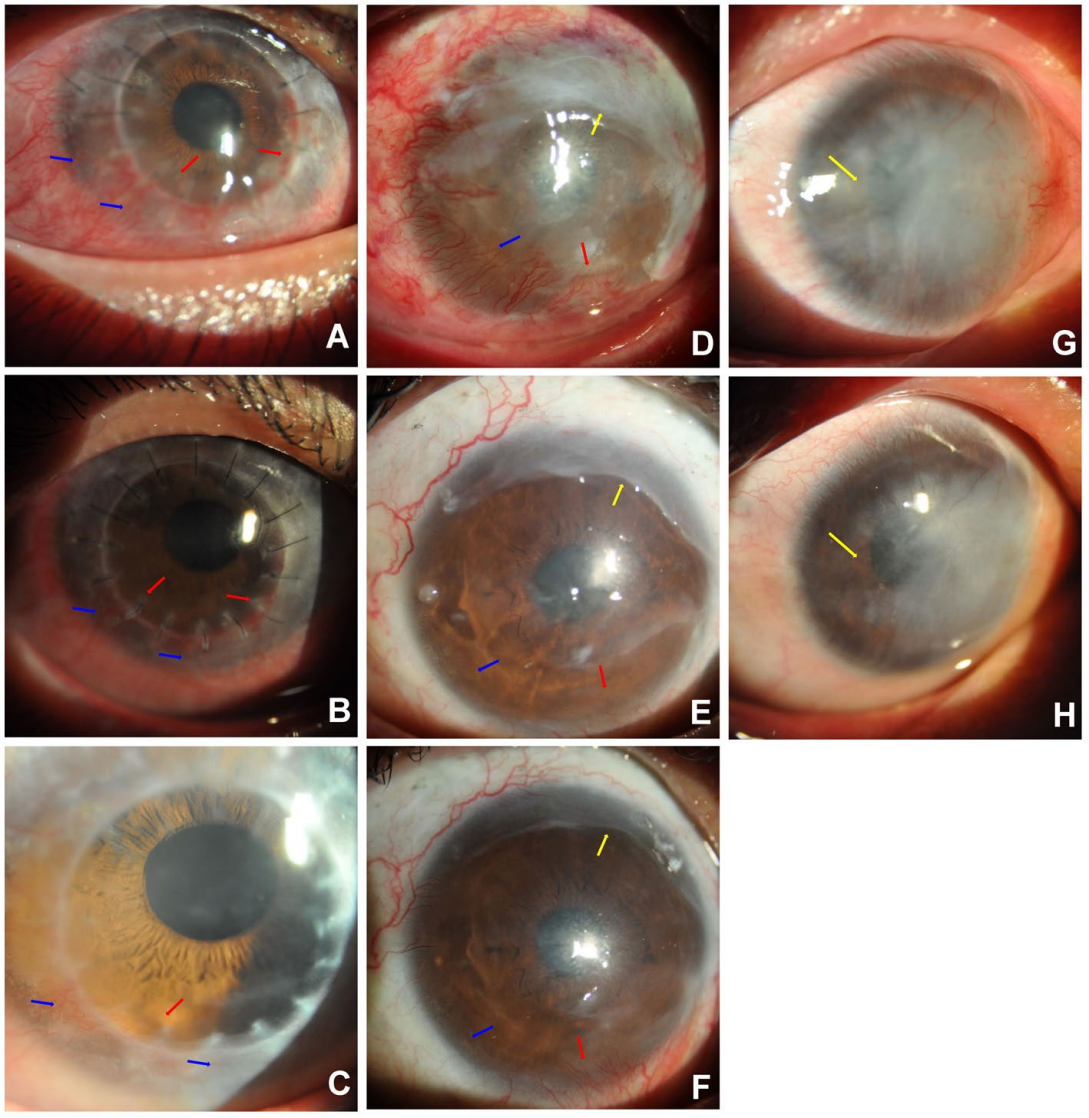
Sample Cases. Case 7 **(A–C)**: Subconjunctival bevacizumab was administered to treat newly formed corneal vessels in the right eye of Case 7. A 19-year-old man with CorNV caused by an alkali burn (lime) had undergone anterior deep lamellar keratoplasty of the central cornea 20 days before presentation. The newly formed superficial vessels encroached onto the graft from the 3 to 9 o’ clock positions [blue arrows, **(A)**], and stromal vessels entered the interface of the lamellar graft and host native cornea [red arrows, **(A)**]. After the first injection, the new superficial corneal vessels were significantly narrower at four weeks [blue arrows, **(B)**], and only a small stromal vessel at 7 o’ clock remained at 12 months post-treatment [red arrow, **(C)**]. Case 10 **(D–F)**: Subconjunctival bevacizumab was administered to treat newly developed CorNV in the right eye of Case 10. A 47-year-old man who had an alkali burn (lime) two months prior and amniotic membrane transplantation 11 days before presented with newly formed superficial CorNV (blue arrow) from the 5 to 11 o’ clock position and residual amniotic membrane (yellow arrow) on the superior cornea **(D)**. The corneal vessels were considerably regressed (blue arrow) at 4 weeks **(E)** and 12 months **(F)** after treatment. The vessels in the fibrovascular membrane at the nasal-inferior cornea became narrower [red arrows, **(E,F)**]. Case 2 **(G,H)**: Alleviation of corneal lipid deposition in Case 2 after bevacizumab injection. The right eye of a 48-year-old man with a history of acid burns (sulfuric) 22 months before showed stable corneal vessels with dense lipid deposition in the horizontal middle cornea [yellow arrow, **(G)**]. The iris could not be observed using a slit–lamp microscope. Six weeks after the subconjunctival bevacizumab injection, corneal lipid deposition was slightly alleviated [yellow arrow, **(H)**], and the iris was observed.

In this study, six patients had corneal neovascular vessels (with fibrovascular membranes) that were more than 1 month old. The large vessels were slightly shorter and narrower than before treatment in Case 7 (Figures 3A–C). Meanwhile, the number of quadrants involved in the CorNV did not change. Most of the fine neovascular vessels, which could previously be identified with slit-lamp microscopy, became invisible after treatment as in Case 10 (Figures 3D–F). In Case 2, corneal lipid deposition was slightly lower after bevacizumab treatment (Figures 3G,H).

## Complications

In Case 1, the corneal epithelial defect areas did not increase or improve after bevacizumab treatment. Filamentary keratitis was observed after treatment in Cases 2, 3, and 9. After the removal of the small white filaments, there was no relapse of filamentary keratitis. In Case 2, an inferior corneal epithelial defect was noted 4 weeks after injection. Trichiasis of the upper eyelid was noted in this patient. The epithelial defect healed after the trichiasis was cleared, the patient wore a therapeutic corneal contact lens (Purevision^®^, Bausch & Lomb Incorporated, Rochester, NY, United States), and the tobramycin/dexamethasone eye drops were terminated.

All patients, except Case 4, had normal IOP during follow-up. The IOP of Case 4’s left eye was 35.7 mmHg (measured using non-contact tonometry) 2 weeks after treatment. After discontinuation of topical tobramycin/dexamethasone eye drops for 4 weeks, the IOP decreased to 11.7 mmHg. No systemic adverse events were observed.

## Discussion

This study has shown that bevacizumab treatment could reduce the length and diameter of neovascular vessels that develop secondary to corneal burns. The peak effect on CorNV occurred four weeks after bevacizumab treatment. A probable reason for this is that the bevacizumab concentration in the normal cornea stroma peaked for 14 days and began to decline at 21 days after the subconjunctival injection (17).

The most interesting finding in this study was that subconjunctival administration of bevacizumab successfully reduced newly formed CorNV. This is in alignment with the findings of other studies. Partial regression of CorNV after subconjunctival injection of bevacizumab was also observed in a rabbit limbal insufficiency model (18), in a rabbit alkali burn-induced CorNV model (19), and in high-risk transplanted corneas (20).

Studies reported that bevacizumab-treatment was less effective on mature/established vessels than on recent onset CorNV in animal (18, 21), human (22–24), and suggested that early bevacizumab application and/or preconditioning can be beneficial to reduce CorNV (25–27). Topical bevacizumab had been reduced in vessel caliber, but with no effect on the area of corneal involvement (28). This absence of vessel regression following topical treatment may be the result of poor penetration of bevacizumab into the eye (29, 30). Reports showed an increase in bioavailability of bevacizumab by subconjunctival injection than topical (23, 26, 27, 31). In general, drug injected into subconjunctival space has two fates: direct transscleral delivery into intraocular tissues or clearance via conjunctival blood and lymphatic flow (32, 33). Since the conjunctival blood vessels do not form a tight junction barrier (34). Bevacizumab subconjunctival injection that directly permeated the sclera and was introduced into the blood circulation and the lymphatic vessels via convective transport with lymphatic fluid (35). Data also showed that most of the bevacizumab was derived from the systemic circulation, and corneal deposition of bevacizumab was observed after subconjunctival injection. Subconjunctival bevacizumab may be a promising treatment for CorNV (29).

Cursiefen et al. found that more than 80% of new vessels were covered by pericytes within 2 weeks of CorNV onset (18). Therefore, the optimal time to administer bevacizumab to induce regression of the immature neovascular vessels is no later than when pericyte coverage occurs. However, the effects of bevacizumab on CorNV with fibrovascular tissues, such as primary (36), recurrent (11), or impending recurrent pterygium (37), are limited. In these situations, topical use of bevacizumab could only partially and transiently decrease conjunctival vascularization (37), not CorNV (11). A recent review, without direct evidence, recommended early treatment with anti-angiogenic agents for preventing CorNV and formation of fibrovascular pannus in patients with chemical burns (38). The results presented here and in our previous report (39) support this recommendation. In our previous report, early application of subconjunctival bevacizumab significantly prevented CorNV after sclerocorneal lamellar keratoplasty for chemical burns (39).

Steroid have been used to treat CorNV on animal studies and in clinical, as it is assumed to be secondary to inflammation. However, steroid is not an ideal drug, as they can cause the replication of pathogens such as fungus and herpes simplex virus and retard corneal wound healing (40–42), and bring complications such as cataract and glaucoma. Besides, corticosteroids have little or even no effect on capillary growth in diseases related to corneal hypoxia or deficiency of limbal cells, especially in severe late-stage ocular chemical burns (19).

In this study, CorNV that developed within a month mostly regressed after treatment. However, vessels with fibrovascular membranes (that formed more than 1 month ago) were observed to be narrower and shorter compared to pretreatment. Although the improvement in these older cases of CorNV was statistically significant, it may not be clinically significant because the quadrants with CorNV involvement were unchanged. Therefore, the risk of corneal graft rejection remains high. Moreover, it was noted that bevacizumab treatment did not inhibit pseudopterygium growth; however, it did reduce the severity of CorNV with fibrovascular membrane formation. As demonstrated in previous reports, alleviation of lipid deposition may contribute to narrowing of large vessels (8) and reduced vascular leakage (43–45) after bevacizumab treatment.

An increase in blood pressure is commonly reported after systemic administration of bevacizumab for rectal cancer (46). However, no systemic events associated with topical eye drops and subconjunctival injection of bevacizumab have been reported (8), and none were observed in this study. Punctuate epithelial erosion (47), new corneal epithelial defects (48), and corneal thinning have been reported in the second month after topical application of bevacizumab (49). In this study, filamentary keratitis was noted in three patients and a new epithelial defect in one patient. Filamentary keratitis may be related to ocular surface instability, and the new epithelial defect was attributed to eyelid trichiasis rather than an adverse effect of bevacizumab, which is consistent with the findings of a previous study (50). It has yet to be determined whether injected bevacizumab is toxic to the corneal epithelium. Various conflicting results have been reported in different *in vitro* (51, 52), and *in vivo* animal studies (53, 54), as well as in human applications (48).

The main drawbacks of this study were the lack of uniformity among the enrolled patients, the small sample size, and the follow-up period being insufficient for long-term evaluation. However, there has been no report to date on the behavioral differences between stromal and superficial corneal vessels. Well-controlled prospective clinical trials are required to determine the optimal dose, route of administration, frequency of use, and possible side effects of this treatment. An innovative method for visualizing and quantifying both superficial and deep corneal neovascular vessels is needed to capture the effects on all corneal vessels (55).

In conclusion, this study has shown that subconjunctival injection of bevacizumab could decrease the severity of CorNV that develops secondary to chemical burns, especially in newly formed vascular vessels. It is recommended that bevacizumab be applied as soon as possible after stabilization of ocular surface epithelialization, to prevent CorNV in patients with corneal chemical burns. Corneal epithelial defects should be closely monitored, and drugs for corneal epithelialization are necessary.

## Data availability statement

The original contributions presented in the study are included in the article further inquiries can be directed to the corresponding author.

## Ethics statement

The studies involving human participants were reviewed and approved by the study protocol conformed to the tenets of the Declaration of Helsinki and was approved by the medical ethics committee of the Affiliated First Hospital of Guangdong Pharmaceutical University (202263). Informed consent was obtained from all patients before the study began. The patients/participants provided their written informed consent to participate in this study. Written informed consent was obtained from the individual(s) for the publication of any potentially identifiable images or data included in this article.

## Author contributions

S-yZ: conceptualization and manuscript review. W-yP, X-fY, B-BZ, and TZ: data acquisition and/or research execution methodology. W-yP and X-fY: data analysis and manuscript preparation. L-wH: data interpretation and manuscript review. All authors contributed to the article and approved the submitted version.

## Funding

This study was supported by the Fundamental Research Funds of the State Key Laboratory of Ophthalmology (Grant number: 303060202400201119) in China.

## Conflict of interest

The authors declare that the research was conducted in the absence of any commercial or financial relationships that could be construed as a potential conflict of interest.

## Publisher’s note

All claims expressed in this article are solely those of the authors and do not necessarily represent those of their affiliated organizations, or those of the publisher, the editors and the reviewers. Any product that may be evaluated in this article, or claim that may be made by its manufacturer, is not guaranteed or endorsed by the publisher.
